# Inhibiting miR-200a-3p Increases Sirtuin 1 and Mitigates Kidney Injury in a Tubular Cell Model of Diabetes and Hypertension-Related Renal Damage

**DOI:** 10.3390/biom15070995

**Published:** 2025-07-11

**Authors:** Olga Martinez-Arroyo, Ana Flores-Chova, Marta Mendez-Debaets, Laia Garcia-Ferran, Lesley Escrivá, Maria Jose Forner, Josep Redón, Raquel Cortes, Ana Ortega

**Affiliations:** 1 Cardiometabolic and Renal Risk Research Group, Biomedical Research Institute of Hospital Clinico de Valencia INCLIVA, 46010 Valencia, Spain; omartinez@incliva.es (O.M.-A.); aflores@incliva.es (A.F.-C.); mmendez@incliva.es (M.M.-D.); laigarfe@alumni.uv.es (L.G.-F.); lescriva@incliva.es (L.E.); maria.jose.forner@uv.es (M.J.F.); josep.redon@uv.es (J.R.); 2 Internal Medicine Unit, Hospital Clinico Universitario, 46010 Valencia, Spain; 3 Department of Medicine, Faculty of Medicine, University of Valencia, 46010 Valencia, Spain; 4 CIBEROBN (CIBER of Obesity and Nutrition Physiopathology), Carlos III Health Institute), 28029 Madrid, Spain; 5 CIBERCV (CIBER of Cardiovascular Diseases), Carlos III Health Institute, 28029 Madrid, Spain

**Keywords:** biomarker, miR-200a-3p, renal damage, Sirtuin 1, tubular injury, extracellular vesicles

## Abstract

Hypertension and diabetes mellitus are key contributors to kidney damage, with the renal tubule playing a central role in the progression of kidney disease. MicroRNAs have important regulatory roles in renal injury and are among the most abundant cargos within extracellular vesicles (EVs), emerging as novel kidney disease biomarkers and therapeutic tools. Previously, we identified miR-200a-3p and its target SIRT1 as having a potential role in kidney injury. We aimed to evaluate miR-200a-3p levels in EVs from patient’s urine and delve into its function in causing tubular injury. We quantified miR-200a-3p urinary EV levels in hypertensive patients with and without diabetes (*n* = 69), 42 of which were with increased urinary albumin excretion (UAE). We analysed miR-200a-3p levels in EVs and cellular pellets, as well as their targets at mRNA and protein levels in renal tubule cells (RPTECs) subjected to high glucose and Angiotensin II treatments, and observed their influence on apoptosis, RPTEC markers and tubular injury markers. We conducted microRNA mimic and inhibitor transfections in treated RPTECs. Our findings revealed elevated miR-200a-3p levels in increased UAE patient urinary EVs, effectively discriminating UAE (AUC of 0.75, *p* = 0.003). In vitro, miR-200a-3p and renal injury markers increased, while RPTEC markers, SIRT1, and apoptosis decreased under treatments. Experiments using miR-200a-3p mimics and inhibitors revealed a significant impact on SIRT1 and decrease in tubular damage through miR-200a-3p inhibition. Increased levels of miR-200a-3p emerge as a potential disease marker, and its inhibition provides a therapeutic target aimed at reducing renal tubular damage linked to hypertension and diabetes.

## 1. Introduction

Highly prevalent diseases such as hypertension (HTN) and diabetes mellitus (DM) are highly related to the development of renal damage, characterised by increases in urinary albumin excretion (UAE) and/or a decrease in the glomerular filtration rate (GFR) [1,2,3]. Alterations in the glomerular filtration barrier and tubule reabsorption processes are behind this injury, in which podocytes and tubular cells are responsible partners. While much attention has been focused on the pathogenesis of glomerular injury, emerging evidence suggests that tubular injury plays a crucial role in the progression of renal damage, placing the tubule not only as a casualty of renal damage but also as a driving force in the development of kidney disease, in which the initial tubular injury triggers the onset of renal fibrosis and tubule-interstitial inflammation by mechanisms not deciphered so far [4]. Therefore, the search for novel targets, mechanisms, and prediction markers of renal injury is the basis for a well based treatment strategy.

In recent years, microRNAs (miRNAs), a subtype of small non-coding RNAs, have garnered considerable interest due to their regulatory roles in various biological processes, including those underlying kidney disease [5]. miRNAs are involved in many renal pathological processes such as inflammation, fibrosis, autophagy, or apoptosis by means of miRNA–mRNA interaction [6,7,8,9]. These non-coding RNAs are one of the most enriched cargos inside extracellular vesicles (EVs) [10], which have arisen as new kidney disease biomarkers and therapeutic tools [11,12,13,14,15]. EVs are small vesicles released by all cell types and found in different body fluids such as plasma and urine [16,17]. These EVs mediate a specific cell-to-cell communication by transferring bioactive molecules on their surface or inside them. Their roles in disease point to the specificity of EV targeting, opening a new interesting field of research based on investigating the EVs’ cargo pathological implications and searching for novel disease biomarkers.

In this sense, Lv et al. reported a pathogenic role of exosomal miR-19b-3p in initiating renal inflammation in mice and high urinary exosome levels of this miRNA in patients with diabetic nephropathy [18]. Moreover, our group has reported the presence of a distinct miRNA signature in urinary and plasma-derived exosomes of hypertensive patients with and without increased UAE [19]; furthermore, we focused on non-coding RNAs circulating in plasma and enclosed in EVs, identifying a profile associated with albuminuria [20]. In addition, we have recently reported an enrichment of fibromiRs and redoximiRs in plasma exosomes from hypertensive and diabetic patients with renal dysfunction. Interestingly, miR-21-5p, a RedoxifibromiR, had significant accuracy for discriminating renal damage in patients [21].

In a previous study, we reported that miR-200a-3p is increased in urine cell pellets of hypertensive and diabetic patients with albuminuria showing an association with increased UAE and a predictive power discriminating renal damage. We also found increased miR-200a-3p levels in podocyte cultures subjected to high glucose and Angiotensin II treatments. Additionally, we obtained interesting results for Sirtuin 1 (SIRT1), a target of miR-200a-3p, being downregulated in patients’ urine pellets and podocyte cultures, also showing a good discriminatory power of increased UAE [22]. Sirtuin 1 is a nicotinamide adenine dinucleotide (NAD+)-dependent deacetylase that has gained attention for its protective effects against renal injury [23]. SIRT1 has been shown to influence claudin 1 (CLDN1) gene expression through epigenetic mechanisms [24]. Claudins are tight junction proteins that in tubules regulate the transepithelial reabsorption of Na+, Cl–, and water [25]. Recent studies further underscore the fundamental role of miR-200a-3p in a wide range of pathological processes. It has been shown to directly target multiple genes involved in apoptosis, inflammation, and oxidative stress pathways [26]. Notably, the functional effects of miR-200a-3p appear to be context-dependent, as emerging evidence highlights its distinct regulatory roles across a range of degenerative and metabolic disorders. For instance, in Alzheimer’s disease, miR-200a-3p promotes β-amyloid-induced neuronal apoptosis by downregulating SIRT1, thereby contributing to neurodegeneration through mechanisms related to oxidative stress and mitochondrial dysfunction [27]. In diabetic cardiomyopathy, miR-200a-3p overexpression mitigates cardiac injury by regulating autophagy via the FOXO3/Mst1/Sirt3/AMPK signalling axis [28]. Furthermore, in diabetic retinopathy, it suppresses cell proliferation and reduces apoptosis by inhibiting the TGF-β2/Smad signalling pathway, highlighting its broader regulatory role in diabetes-related complications [29].

Understanding the intricate interplay between miR-200a-3p and SIRT1 in the context of tubular damage holds promise for the development of novel therapeutic strategies aimed at mitigating renal injury in HTN and diabetes-associated renal damage. With this context, in this study we aimed to delve into the role of miR-200a-3p in renal damage, focusing on the relevance of urinary EVs as potential biomarkers in hypertensive and diabetic patients with albuminuria and in their involvement in tubular injury through in vitro studies.

## 2. Materials and Methods

### 2.1. Study Population

This study included 69 Caucasian hypertensive patients with and without DM and/or increased UAE, from the Internal Medicine area of Hospital Clínico Universitario of Valencia (Spain). HTN was defined according to the European Society of Hypertension (systolic blood pressure (SBP) > 140 mmHg and/or diastolic blood pressure (DBP) < 90 mmHg) [30], and DM was defined according to the World Health Organization [31]. Among all patients, 42 showed increased UAE (UAE group). For the analyses, we also divided the cohort of patients into four groups: 9 patients with DM (DM Non-UAE), 27 patients with DM and increased UAE (DM UAE), 19 non-diabetic patients (Non-DM Non-UAE), and 14 non-diabetic patients with increased UAE (Non-DM UAE). All patients received antihypertensive treatment, and DM subjects were treated with oral antidiabetic agents.

### 2.2. Urine Sample Processing and UAE Data Measurements

Fresh first morning urine (200 mL) was collected from patients in sterile containers. All samples were processed within one hour after reception. First, samples were centrifuged at 2250× *g* for 30 min at 4 °C to precipitate cells and debris. Then, EV isolation from cell-free urine was performed as described below. Urinary albumin excretion (UAE) was assessed using a nephelometric immunoassay (Behring nephelometer (Behring Institute, Dresden, Germany)). For each subject, albuminuria was determined as the average of morning spot urine samples and reported as the albumin-to-creatinine ratio (ACR), expressed in mg/g. Increased UAE was defined as ACR ≥ 30 mg/g.

### 2.3. Renal Proximal Tubular Epithelial Cell (RPTEC) Culture and Treatment

Conditionally immortalised human renal proximal tubule epithelial cell line (RPTEC/TERT1) was obtained from Evercyte (Evercyte GmbH, Vienna, Austria). This cell line was created from human kidney cortex and transduced with retrovirus carrying the catalytic subunit of human telomerase (hTERT) [32]. RPTECs present a cobblestone morphology, are capable of formation of domes and tight junctions, and express cell-type-specific markers. Briefly, RPTECs were cultured in proportion 1 to 1 of Dulbecco’s Modified Eagle Medium (DMEM) and Ham’s F/12 supplemented with 10% exosome-depleted foetal bovine serum (FBS) (all from Biowest, Nuaillé, France), 5 µg/mL of insulin-transferrin-selenium (ITS), 2 mM of GlutaMAX (both from Gibco, Thermo-Fisher Scientific, Waltham, MA, USA), 36 ng/mL of hydrocortisone, 10 ng/mL human epidermal growth factor (hEGF) (both from Sigma-Aldrich, St. Louis, MO, USA), and 1% penicillin/streptomycin (P/S, Biowest, Nuaillé, France). Cells were cultured at 37 °C until reaching 80% confluence and then replated at 70% confluence to induce differentiation. Subsequently, RPTECs were differentiated for 12 days at 37 °C in the absence of FBS. After differentiation, RPTECs were treated with glucose and Angiotensin II (Ang II). For glucose treatment, D-(+)-Glucose (Sigma Aldrich, St. Louis, MO, USA) was added at normal (NG; 5.5 mM) and high (HG; 30 mM) concentrations, and cells were incubated for 48 and 72 h at 37 °C. For Ang II treatment, cells were incubated with 1 µM Ang II (Sigma Aldrich, St. Louis, MO, USA) for 24 and 48 h at 37 °C depending on the experiment performed.

### 2.4. Cell Apoptosis Analysis

Apoptosis of treated RPTECs was measured by flow cytometry. After treatment with glucose and Ang II, cell pellets were treated with trypsin, resuspended, centrifuged at 1300 rpm for 3 min, and washed. They were subsequently resuspended in 200 µL of annexin V binding buffer (Immunostep, Salamanca, Spain) and incubated in the dark with 5 µL of annexin V for 15 min, followed by 7 µL of 7-amino-actinomycin D (7-AAD) for 5 min. Finally, 400 µL of annexin binding buffer was added. Flow cytometry analysis was performed on 10,000 events per replicate using a BD LSRFortessa X-20 cytometer and FACSDiva 8.0.1 software (BD Biosciences, Franklin Lakes, NJ, USA) as previously performed by our group [33].

### 2.5. Transfection of RPTEC with miR-200a-3p Mimic and Inhibitor

RPTECs were transfected with miR-200a-3p mimic (hsa-miR-200a-3p Ambion™ mirVana™ miRNA Mimic ID:MC10991; Thermo Fisher Scientific, Waltham, MA, USA) and miR-200a-3p inhibitor (hsa-miR-200a-3p Ambion™ mirVana™ miRNA Inhibitor ID: MH10991; Thermo Fisher Scientific, Waltham, MA, USA) using Lipofectamine 3000 (Invitrogen, Thermo Fisher Scientific, Waltham, MA, USA) using Opti-MEM^®^ (Invitrogen, Thermo Fisher Scientific, Waltham, MA, USA), the appropriate negative controls for mimic and inhibitor transfections (Ambion™ mirVana™ miRNA mimic negative control #1, ID: 4,464,058 and Ambion™ mirVana™ miRNA inhibitor negative control #1, ID: 4464076, from Thermo Fisher Scientific, Waltham, MA, USA) and culture medium without antibiotics, following the manufacturer’s instructions. To monitor transfection efficiency, a Cy3™ dye-labelled negative control was employed (Invitrogen, Thermo Fisher Scientific, Waltham, MA, USA). After 24 h, transfection medium was replaced by fresh DMEM and Ham’s F12 mixture with 1% P/S and ITS. Treatments with glucose and Ang II were performed as described above, and cells were incubated for 24 h and 48 h at 37 °C, respectively.

### 2.6. EV Isolation from RPTEC Cultures and Patients’ Urine

Sequential ultracentrifugation protocol was followed to isolate exosomes from cell culture medium and patients’ urine [19]. Briefly, from the collected medium and urine, cells and debris were removed by centrifugation at 2250× *g* for 30 min. The resulting supernatant, free of cells, was further centrifuged at 20,000× *g* for 45 min using an Optima L-100K ultracentrifuge with a 70 Ti rotor (Beckman Instruments, Brea, CA, USA) to pellet larger extracellular vesicles (EVs). Subsequently, the remaining supernatant was centrifuged at 124,000× *g* for 70 min to collect the exosome-enriched pellet, which, when it belonged to cell culture medium, was resuspended in 0.2 µm filtered phosphate-buffered saline (PBS) and processed for downstream analyses. In the case of EV fractions isolated from patients’ urine, after the resuspension in 0.2 µm filtered PBS, exosomal fraction was transferred into new smaller tubes (13.5 mL), adding 500 μL of dithiothreitol (DTT) (Sigma-Aldrich), to minimise potential disulphide bonds to prevent non-vesicular proteins from binding to EVs, and incubated for 10 min at 37 °C. Thus, 5 mL of 0.2 μm cold filtrated PBS was added, and tubes were lastly ultracentrifuged at 124,000 g for 70 min at 4 °C. Finally, the pellet obtained was resuspended in 0.2 µm filtered PBS and processed for further use.

### 2.7. EV Concentration Assessment

To characterise EV populations, nanoparticle tracking analysis (NTA) was carried out using NanoSight LM10 (Malvern Panalytical Ltd., Malvern, UK). For this, samples were diluted 1/50 with filtered PBS to achieve the recommended concentration (20–120 particles per frame) before being injected into the NanoSight, (405 nm laser and sCMOS camera (Oxford Instruments, OX13 5QX, UK)). Subsequently, NTA software version 3.3 (Dev Build 3.3.104) was used to analyse the obtained data, and the following parameters were set to automatic: Min Track Length, Max Jump Distance, and Blur, with the detection threshold fixed at 5. The camera level was adjusted to 15, and five 30 s recordings at 30 frames per second were captured, with manual monitoring of the temperature.

### 2.8. RNA Isolation and cDNA Synthesis

Total RNA from cell pellets was extracted using the miRNeasy Mini Kit (Qiagen, Hilden, Germany) according to the manufacturer’s protocol. For RNA isolation from exosome samples, the Total Exosome RNA and Protein Isolation Kit (Invitrogen, Thermo Fisher Scientific, Waltham, MA, USA) was employed. RNA concentration and purity were assessed using a NanoDrop 2000 spectrophotometre (Thermo Fisher Scientific, Waltham, MA, USA). Samples were subsequently stored at −80 °C until further use.

For mRNA quantification, cDNA was synthetized using the Ready-To-Go You-Prime First-Strand Beads kit (Cytiva, Barcelona, Spain) following the protocol indications. For miRNA analysis in cellular pellet and exosomes derived from cell culture media, 5 μL of total RNA was used in the TaqMan™ miRNA reverse transcription Kit (Applied Biosystems, Foster City, CA, USA) according to the supplier’s instructions. For miRNA analysis in exosomes derived from patients’ urine (EXO-U), we employed the TaqMan™ Advanced miRNA cDNA Synthesis Kit (Applied Biosystems, Foster City, CA, USA) according to the supplier’s protocol. The resulting cDNA was stored at −20 °C until further use.

### 2.9. Quantitative Real-Time Polymerase Chain Reaction (RT-qPCR)

Quantitative Real-Time Polymerase Chain Reaction (RT-qPCR) was performed in the LightCycler 480 II real-time PCR system (Roche, Mannheim, Germany). Specific primers were designed by Primer 3 software for mRNA analyses (Table S1). A mix of each primer set with Qiagen Multiplex PCR Master Mix and LC Green reagent (Qiagen, Hilden, Germany) was added to 2 μL of diluted cDNA. For miRNA analyses in cellular pellets and exosomes derived from culture media, a TaqMan™ Small RNA Assay Protocol was followed. Briefly, a mix containing 1.33 μL of diluted cDNA, TaqMan™ Universal Master Mix II (no UNG), and specific TaqMan™ microRNA assay probe for miR-200a-3p (ID: 000502; Applied Biosystems, Foster City, CA, USA) was used. For miR-200a-3p analysis in EXO-U, TaqMan™ Fast Advanced Master Mix, already prepared to be used with Taqman™ microRNA Assay probe for miR-200a-3p (PCR universal master mix II, Applied Biosystems), was used combining 7 μL of Master Mix, 0.5 μL of each specific TaqMan™ probe, and 2.5 μL of 1/10 diluted cDNA.

All assays were performed in triplicate, including appropriate controls and a non-template control. Additionally, melting curve analysis was conducted to assess the specificity of the generated amplicons. β-Actin (ACTB) and β2-microglobulin (B2MG) served as housekeeping genes for mRNA normalisation, while cel-miR-39-3p (ID: 000200) was used as an external reference for miRNA analysis. The relative expression of each target gene or miRNA was calculated using the 2^−ΔΔCt^ comparative method (where Ct represents the threshold cycle). Results were expressed as fold change (FC), comparing the target gene levels to the average expression of the housekeeping genes.

### 2.10. Homogenization of Samples, Gel Electrophoresis, and Western Blot Analyses

RPTEC pellet and exosome samples from cells and patients’ urine were lysed using RIPA buffer (Thermo Fisher Scientific, Waltham, MA, USA) supplemented with a protease inhibitor cocktail (Sigma-Aldrich, St. Louis, MO, USA). The supernatant was collected after centrifugation at 15,000× *g* for 15 min at 4 °C. Protein concentration was determined by the Lowry method using bovine serum albumin (BSA) as the standard. Protein samples were separated on NuPAGE 4–12% polyacrylamide gels (Invitrogen, Carlsbad, CA, USA) and transferred onto polyvinylidene difluoride (PVDF) membranes. Membranes were blocked overnight and then incubated with primary antibodies for 2 h at room temperature. The primary antibodies used were as follows: rabbit monoclonal anti-e-cadherin (1/1000, Thermo Fisher Scientific, Waltham, MA, USA), rabbit polyclonal anti-aquaporin-1 (1/500, EMD Millipore, Burlington, MA, USA), rabbit monoclonal anti-sirt-1 (1/3000, Abcam, Cambridge, UK), rabbit monoclonal anti-claudin-1 (1/2000, Abcam, Cambridge, UK), rabbit polyclonal anti-KIM-1 (1/500, Abcam, Cambridge, UK), rabbit polyclonal anti-IL-18 (1/1000, Abcam, Cambridge, UK), and rabbit polyclonal anti-synaptopodin (1/1000, Sigma-Aldrich, St. Louis, MO, USA). For characterisation of EV isolated fractions, we used rabbit monoclonal anti-CD9 (1/1000, Abcam, Cambridge, UK), mouse monoclonal anti-syntenin (1/500, OriGene, Rockville, MD, USA), mouse monoclonal anti-CD63 (1/200, Abcam, Cambridge, UK), rabbit monoclonal anti-calnexin (1/1000, Abcam, Cambridge, UK), rabbit monoclonal anti-GM130 (1/1200, Abcam, Cambridge, UK), and rabbit polyclonal anti-Nup62 (1/600, Abcam, Cambridge, UK). Mouse monoclonal anti-β-actin antibody (1:6000, Sigma-Aldrich, St. Louis, MO, USA) was employed as a loading control. Following washes with Tris-buffered saline containing 0.1% Tween 20 (TBS-T; 20 mM Tris-HCl, 150 mM NaCl), membranes were incubated at room temperature for 1 h with alkaline phosphatase-conjugated anti-rabbit IgG or anti-mouse IgG secondary antibodies (Sigma-Aldrich, St. Louis, MO, USA). Subsequently, membranes were washed three times with TBS-T and TBS, and antibody binding was visualised using the chromogen 5-bromo-4-chloro-3-indolyl phosphate/nitro blue tetrazolium (BCIP/NBT, Sigma-Aldrich, St. Louis, MO, USA). Finally, bands were digitised and quantified using ImageQuant™ 7.0 TL software.

### 2.11. Statistical Analysis

Data are expressed as FC in text for mRNA, miRNA, and protein levels and FC and mean  ±  standard error of the mean (SEM) in graphs. The Kolmogorov–Smirnov test was applied to assess the normality of variables. Comparisons between two groups were performed using either Student’s *t*-test or the Mann–Whitney U test, depending on the data distribution. Pearson’s and Spearman’s correlation coefficients (depending on data distribution) were calculated to analyse the association between variables. The sensitivity, specificity, and predictive value of miR-200a-3p were calculated by generating Receiver Operating Characteristic (ROC) curves, with calculation of the area under the curve (AUC) and with 95% confidence interval (CI). A significance level of *p* < 0.05 was established. Statistical analyses were conducted using SPSS software (version 20), and graphical representations were generated with GraphPad Prism (version 9.00).

## 3. Results

### 3.1. miR-200a-3p Expression in Patients’ Urinary Exosomes

First, we reported the clinical characteristics of the patients and the differences between study groups in Table 1. Briefly, patients with DM and increased UAE were older, had an increased body mass index, glucose levels, glycated haemoglobin and albuminuria levels. Then, we sought to determine the expression of miR-200a-3p on exosomes isolated from patients’ urine (EXO-U), as we had found interesting alterations and associations of this miRNA in previous studies of our group.

**Figure 1:**
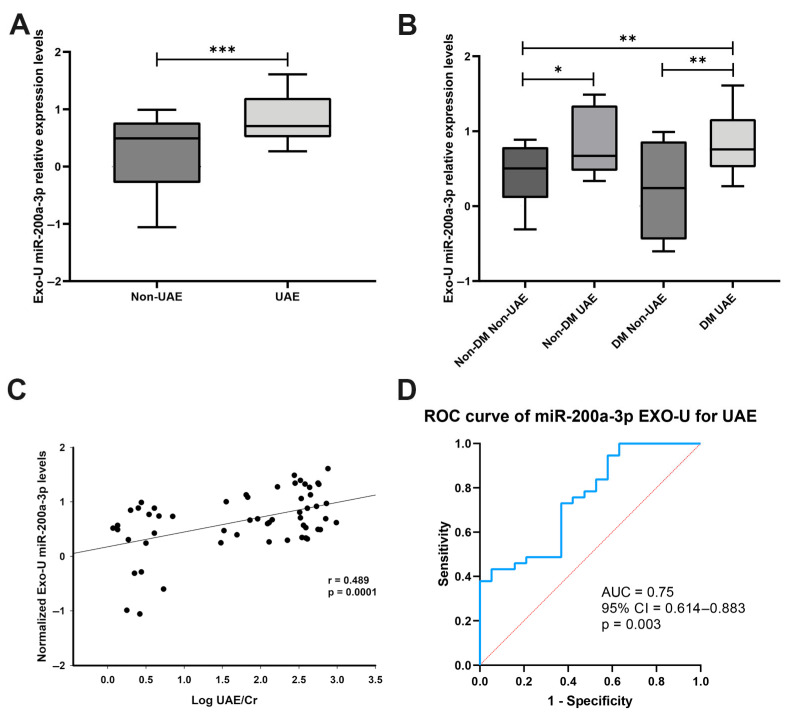
miR-200a-3p analysis in EXO-U samples from patients: (**A**). Box plots of EXO-U miR-200a-3p levels in UAE versus non-UAE groups. (**B**). Box plots of EXO-U miR-200a-3p levels analysed in four groups: Non-DM Non-UAE, Non-DM UAE, DM Non-UAE and DM UAE. (**C**). Association between EXO-U miR-200a-3p levels with UAE/Cr in patients. (**D**). ROC curves are constructed using the EXO-U miR-200a-3p levels for predicting UAE. The AUC and 95% CI are computed and are shown for the ROC curve; Blue: ROC curve; red: reference line (AUC=0.5). DM: diabetes mellitus; EXO-U: exosome from urine; ROC: Receiver Operator Characteristic; and UAE: urinary albumin excretion. * *p* < 0.05; ** *p* < 0.01 and *** *p* < 0.001.

In exosomes obtained from patients’ urine, we found that miR-200a-3p was increased in the UAE group versus the non-albuminuric group (Non-UAE), (2.69 FC, *p* < 0.01; Figure 1A). When we stratified the study patients in four groups, we also observed increases in miR-200a-3p in Non-DM UAE patients (2.72 FC, *p* < 0.05 vs. Non-DM Non-UAE) and DM UAE patients when compared to both the Non-DM, Non-UAE group (2.76 FC, *p* < 0.01) and to the DM Non-UAE group (2.82 FC, *p* < 0.01) (Figure 1B). We further analysed the associations between the UAE levels (log UAE/creatinine) and the EXO-U miR-200a-3p levels, and we found a positive correlation, indicating that increased UAE is accompanied by high levels of the miRNA (r = 0.489, *p* < 0.0001; Figure 1C). In addition, we constructed a ROC curve to assess the predictive capacity of EXO-U miR-200a-3p for discriminating the presence of UAE. This analysis showed an AUC of 0.75 (95% CI 0.614–0.883, *p* = 0.003; Figure 1D).

### 3.2. Cell Line and EV Characterisation

RPTEC line was characterised to show typical features such as morphology and dome formation by optical microscopy (Figure S1A) and tubular markers through Western blot in which we compared the expression between RPTEC and podocyte markers, showing the specificity of the cell line analysed (Figure S1B). EVs obtained from the RPTEC medium supernatant were characterised analysing their size, concentration, and purity. NTA showed a main peak of small EV population (140–200 nm, exosomal fraction; Figure S1C). Western blot confirmed the purity of the EVs, with vesicles enriched in the surface and cytoplasmic EV markers CD9, syntenin and CD63, and the absence of intracellular compartment markers, present only in the cell pellet (Figure S1D). These analyses evidence that the isolation and purification of EVs were correctly performed, resulting in an enriched EV pellet predominantly composed of exosomes.

### 3.3. Effect of Treatments on RPTECs

**Figure 2:**
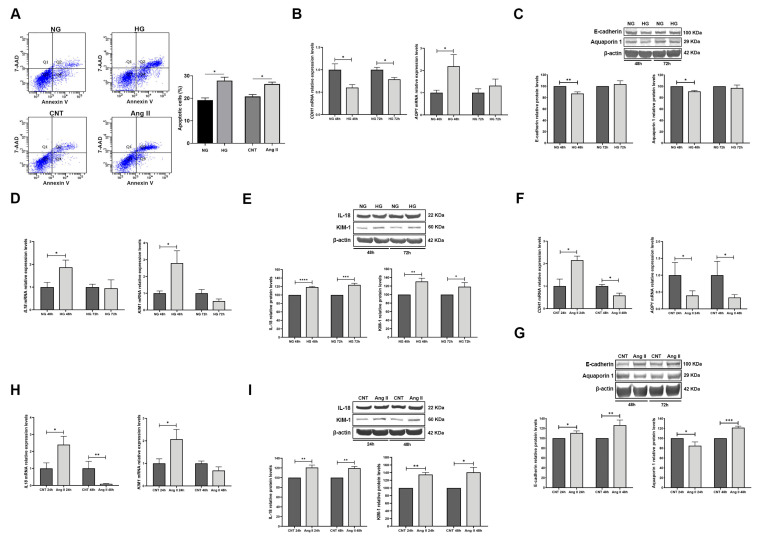
Effect of treatments with HG and Ang II on RPTECs: (**A**) Apoptosis of RPTECs assessed by flow cytometry. Images show cells double-stained with annexin V/-amino-actinomycin (7-AAD). Quadrant 1 (Q1) shows cells with lost intact cell membranes that bind 7-AAD and exclude annexin V. Quadrant 2 (Q2) represents cells with advanced stages of apoptosis or necrosis that are both annexin V- and 7-AAD-positive. Quadrant 3 (Q3) shows viable cells that did not bind annexin V or 7-AAD. Quadrant 4 (Q4) depicts early apoptotic cells, positive for annexin V but with still intact cell membranes (7-AAD-negative). Graphs show apoptosis percentages in glucose and Ang II-treated RPTECs. (**B**) mRNA levels of RPTEC markers CDH1 and AQP1 under NG and HG conditions. (**C**) Western blot membranes and graphs showing protein levels of E-Cadherin and Aquaporin 1 under HG conditions. (**D**) mRNA levels of tubular injury markers IL18 and KIM1 under NG and HG conditions. (**E**) Western blot membranes and graphs showing protein levels of IL-18 and KIM-1 under HG conditions (**F**) mRNA levels of RPTEC markers CDH1 and AQP1, under Ang II treatment (**G**). Western blot membranes and graphs showing protein levels of E-Cadherin and Aquaporin 1 under Ang II treatment. (**H**). mRNA levels of tubular injury markers IL18 and KIM1 under Ang II treatment. (**I**) Western blot membranes and graphs showing protein levels of IL-18 and KIM-1 under Ang II treatment. Ang II: Angiotensin II; CNT: control; NG: normal glucose; and HG: high glucose. (Original images can be found in Figure S2) Data are shown as mean ± SEM (standard error of the mean). *N* = 5 biological replicates for each group. For the NG and CNT groups, mRNA levels are normalised to 1, and protein levels are normalised to 100. * *p* < 0.05, ** *p* < 0.01, *** *p* < 0.001 and **** *p* < 0.0001 vs. the NG or CNT group.

Apoptosis assays showed that HG and Ang II treatments induced 27.8% and 26.3% of apoptosis, respectively (*p* < 0.05 for both treatments; Figure 2A). Next, we analysed the mRNA and protein levels of the RPTECs and injury markers under glucose and Ang II treatments. Under HG conditions, CDH1 (e-cadherin) mRNA levels were decreased at 48 h and 72 h of treatment duration (−1.66 FC, −1.27 FC, respectively, *p* < 0.05 for both). For AQP1 (Aquaporin 1) mRNA levels, we found an increase at 48 h of treatment (2.34 FC, *p* < 0.05) (Figure 2B). Protein levels of these markers also evidenced decreased levels at 48 h of treatment (E-cadherin −1.15 FC, *p* < 0.01; Aquaporin 1 −1.10 FC, *p* < 0.05; Figure 2C). In addition, tubular injury markers KIM1 and IL18 showed increases under HG at mRNA levels only at 48 h of treatment (2.80 FC, 1.87 FC, *p* < 0.05 for both; Figure 2D). Similar results were obtained for their protein levels, with increased levels of both injury markers at both treatment times (1.19 FC, *p* < 0.0001, 48 h; 1.24 FC, *p* < 0.001, 72 h, for IL-18; and 1.31, *p* < 0.01, 48 h, 1.23 FC, *p* < 0.05, 72 h, for KIM-1; Figure 2E).

Regarding Ang II treatments, we observed that mRNA levels of CDH1 were increased at 48 h after treatment (2.15 FC, *p* < 0.05) but decreased after 72 h (−1.71 FC, *p* < 0.05). AQP1 showed a decrease at 48 h and 72 h (−2.52 FC, −2.96 FC, respectively, *p* < 0.05) (Figure 2F). The protein levels of E-cadherin at both 48 h and 72 h treatments were increased (1.11 FC, *p* < 0.05 and 1.26 FC, *p* < 0.01, respectively). Meanwhile, Aquaporin 1 displayed different trends between treatment times (−1.25 FC *p* < 0.05 at 48 h and 1.20 FC, *p* < 0.001 at 72 h) (Figure 2G). As well, tubular damage was evidenced by an augmentation of KIM1 and IL18 mRNA levels at 48 h (2.07 FC and 2.40 FC, *p* < 0.05 for both). But, interestingly we observed a decrease after 72 h, only significant in the case of IL18 (−10.75 FC, *p* < 0.01) (Figure 2H). As well, protein levels of both injury markers showed an increase at both treatment times (1.21 FC, 24 h and 1.20 FC, 48 h, *p* < 0.01 for both, for IL-18; 1.35, *p* < 0.01, 24 h, 1.41 FC, *p* < 0.05, 48 h, for KIM-1; Figure 2I).

### 3.4. miR-200a-3p Expression in Treated RPTECs, Exosomes, and Target Analyses

**Figure 3:**
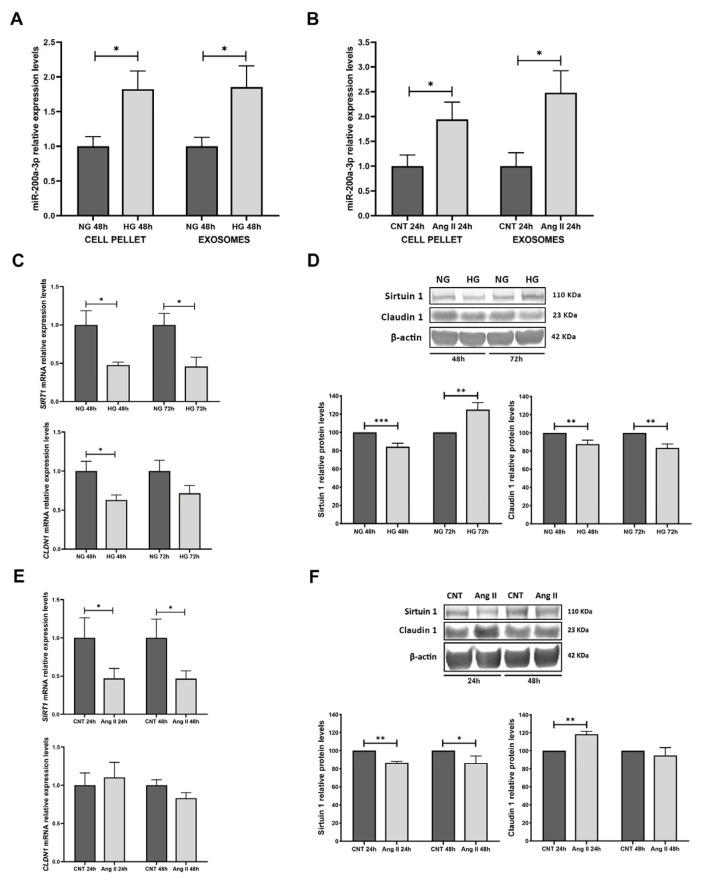
miR-200a-3p analysis in RPTEC cultures: (**A**). miR-200a-3p levels in cell pellet and exosomes from RPTEC cultures under glucose treatment. (**B**). miR-200a-3p levels in cell pellet and exosomes from RPTEC cultures under Ang II treatment. (**C**). mRNA levels of SIRT1 and CLDN1 under glucose treatments. (**D**). Western blot membranes and graphs showing protein levels of Sirtuin 1 and Claudin 1 under HG conditions. (**E**). mRNA levels of SIRT1 and CLDN1 under Ang II treatments. (**F**). Western blot membranes and graphs showing protein levels of Sirtuin 1 and Claudin 1 under Ang II treatment. Ang II. Angiotensin II; (Original images can be found in Figure S3) CNT: control. N = 5 biological replicates for each group. Ang II: Angiotensin II; CNT: control; NG: normal glucose; HG: high glucose. For the NG and CNT groups, mRNA and miRNA levels are normalised to 1, and protein levels are normalised to 100. * *p* < 0.05; ** *p* < 0.01 and *** *p* < 0.001 vs. the NG or CNT group.

We analysed the miRNA levels of miR-200a-3p in cellular pellets and exosomes from RPTECs subjected to HG and Ang II treatments. We found that, under HG, miR-200a-3p was increased in the cell pellet (1.82 FC, *p* < 0.05) and exosomes derived from cell culture media (1.85 FC, *p* < 0.05) (Figure 3A). When RPTECs were treated with Ang II, we found that miR-200a-3p was increased in cellular pellets (1.94 FC, *p* < 0.05) and also in the isolated exosomes (2.48 FC, *p* < 0.05) (Figure 3B).

We further analysed the miR-200a-3p target SIRT1 mRNA levels and their associated molecule CLDN1 (Claudin 1) under glucose and Ang II treatments. Under HG and 48 h of treatment, SIRT1 and CLDN1 had decreased levels (−2.10 FC, −1.59 FC, respectively, *p* < 0.05 for both). After 72 h of treatment, SIRT1 was also decreased (−2.18 FC, *p* < 0.05; CLDN1, trend not significant (n.s)) (Figure 3C). When we analysed the protein levels under glucose treatment, Sirtuin 1 and Claudin 1 displayed a general diminution under 48 h treatment (−1.19 FC, *p* < 0.001 and −1.14 FC, *p* < 0.01, respectively), also evident for Claudin 1 at 72 h (−1.20 FC, *p* < 0.01). Conversely, after 72 h, Sirtuin 1 levels were increased (1.25 FC, *p* < 0.01) (Figure 3D).

Under Ang II treatment, we observed that mRNA levels of SIRT1 showed a decrease at 48 h −2.13 FC, *p* < 0.05) and also after 72 h (−2.14 FC, *p* < 0.05). In contrast, CLDN1 did not show a significant change (Figure 3E). At the protein level, we observed that Sirtuin 1 had a significant decrease in both time treatments (−1.16 FC for both, *p* < 0.01 and *p* < 0.05, respectively). Claudin 1 increased at 48 h (1.18 FC, *p* < 0.01) (Figure 3F).

### 3.5. miR-200a-3p Overexpression and Inhibition Effects on SIRT1 and Tubular Damage

We performed miR-200a-3p mimic and inhibitor experiments to analyse the influence of this miRNA on the expression of its target SIRT1 and the related molecule CLDN1. First, to assure the transfection efficiency, we used Cy3 labelling, which allowed us to see by fluorescence the internalisation of liposomes with the mimic and inhibitor (Figure S4A). We also quantified the expression of miR-200a-3p with its mimic and inhibitor in combination with glucose and Ang II treatments (Figure S4B). As expected, miR-200a-3p levels increase with mimic without treatment (223.4 FC, *p* < 0.001; 230.95 FC, *p* < 0.05, respectively), also with only HG or Ang II (2.92 FC, *p* < 0.05; 1.46 FC, *p* < 0.05, respectively) and with mimic with HG or Ang II (163.4 FC, *p* < 0.05; 230.9 FC, *p* < 0.05, respectively). Inhibitor experiments exerted the opposite effect, downregulating the expression of miR-200a-3p without any treatments (−2.33 FC, *p* < 0.05; (−1.81 FC, *p* < 0.05, respectively), with an increase in the absence of inhibitor and with HG or Ang II (1.62 FC, *p* < 0.05; 1.25 FC, n.s) and with a downregulation of its levels under inhibitor and HG, which is less potent than without treatment (−1.73 *p* < 0.05) but shows no change in the case of inhibitor and Ang II treatment.

**Figure 4:**
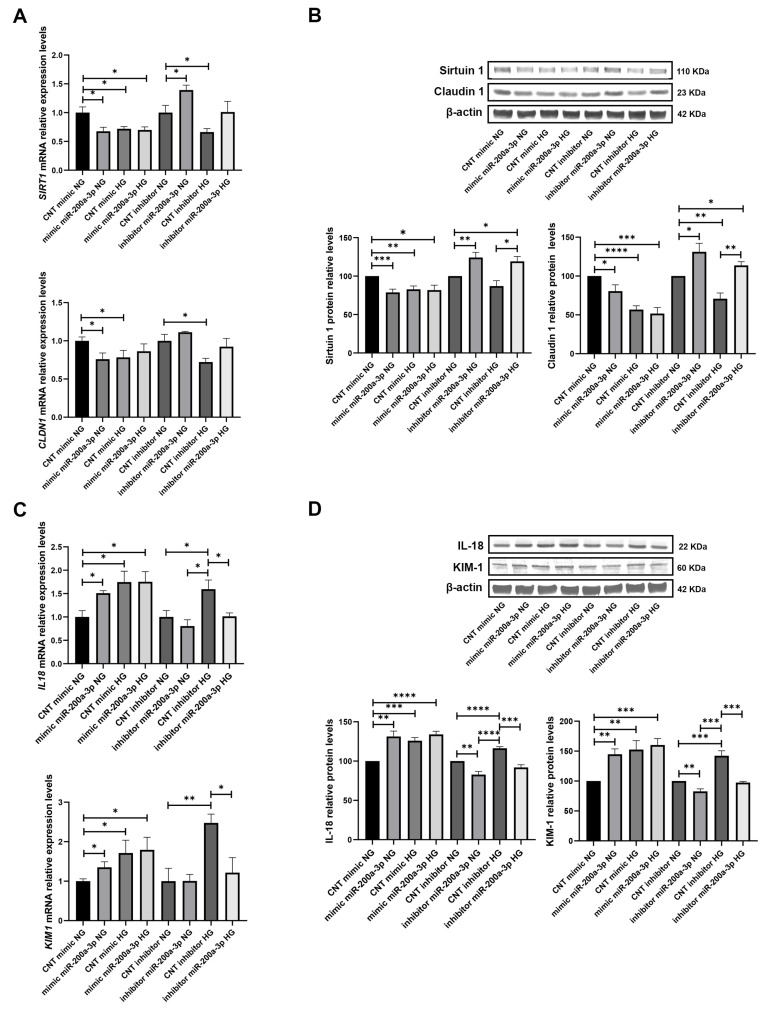
Influence of miR-200a-3p mimic and inhibitor on its miRNA target SIRT1 and in tubular injury under HG treatment: (**A**) SIRT1 and CLDN1 mRNA levels in RPTEC cultures subjected to miR-200a-3p mimic and inhibitor experiments under HG treatment. (**B**) Western blot membranes and graphs showing protein levels of Sirtuin 1 and Claudin 1 under miR-200a-3p mimic and inhibitor and HG conditions. (**C**) Tubular injury markers IL18 and KIM1 mRNA levels in RPTEC cultures subjected to miR-200a-3p mimic and inhibitor experiments under HG. (**D**) Western blot membranes and graphs showing protein levels of IL-18 and KIM-1 under miR-200a-3p mimic and inhibitor and HG conditions. (Original images can be found in Figure S5) N = 5 biological replicates for each group. CNT: control; NG: normal glucose; HG: high glucose. For the CNT mimic NG and CNT inhibitor NG groups, miRNA levels are normalised to 1. * *p* < 0.05; ** *p* < 0.01, *** *p* < 0.001, and **** *p* < 0.0001 vs. the CNT mimic, NG, and CNT inhibitor NG groups.

The analysis of the target SIRT1 with a mimic of miR-200a-3p and glucose treatment displayed a downregulation with a miR-200a-3p mimic (−1.48 FC, *p* < 0.05) and also when treated only with HG (−1.37 FC, *p* < 0.05) and with the mimic and treatment (−1.43 FC, *p* < 0.05). With the inhibitor of miR-200a-3p, we observed that SIRT1 mRNA levels were increased (1.39 FC, *p* < 0.05). Interestingly, the combination of the inhibitor with the treatment produced a compensatory effect between the enhancement of the inhibitor and the downregulation of the treatment in which SIRT1 did not show significant changes (Figure 4A). Similarly, protein levels of Sirtuin1 showed a decrease with the mimic of miR-200a-3p, with the HG treatment and with the combination of both (−1.27 FC, *p* < 0.001; −1.21 FC, *p* < 0.01; −1.22, *p* < 0.05, respectively). As expected, levels were increased with the miR-200a-3p inhibitor, as well as with the addition of HG, evidencing a compensatory effect of the inhibitor (1.24, *p* < 0.01 and 1.19, *p* < 0.05, respectively) (Figure 4B). Furthermore, the mRNA levels of CLDN1 were downregulated in the presence of the mimic and under HG (−1.32, *p* < 0.05 and −1.28, *p* < 0.05, respectively), but no longer effects were observed with the inhibitor (Figure 4A). Its protein levels changed in the same way as observed with Sirtuin 1, with a decrease with the mimic, HG, both conditions (−1.24 FC, *p* < 0.05; −1.76 FC, *p* < 0.0001; −1.94, *p* < 0.001, respectively), and an increase with the inhibitor and inhibitor plus HG (1.31, *p* < 0.05 and 1.14, *p* < 0.05, respectively) (Figure 4B).

We further analysed the levels of the tubular injury markers under the mimic and inhibitor of miR-200a-3p and glucose treatment. IL18 mRNA levels revealed an increase in mimic condition (without HG), HG, and both (1.51 FC, *p* < 0.05; 1.75 FC, *p* < 0.05; and 1.75 FC, *p* < 0.05, respectively) and the levels were decreased to control (CNT) mimic conditions when an inhibitor of miR-200a-3p was used. The same was observed for KIM1 mRNA levels, with increases under the mimic, HG, both (1.35 FC, *p* < 0.05; 1.71 FC, *p* < 0.05; and 1.79 FC, *p* < 0.05, respectively), and a compensation of damage with the inhibitor (Figure 4C). Protein levels of both injury markers followed the same tendency as mRNA levels, with increased protein levels under the mimic, glucose, and both conditions (1.31 FC, *p* < 0.01; 1.26 FC, *p* < 0.001; 1.34, *p* < 0.0001, for IL-18; and 1.45 FC, *p* < 0.01; 1.53 FC, *p* < 0.01; and 1.60, *p* < 0.001, for KIM-1). With the inhibitor, we observed a reduction in injury through the decrease in IL-18 and KIM-1 levels (−1.21, *p* < 0.01 for both injury markers) (Figure 4D).

**Figure 5:**
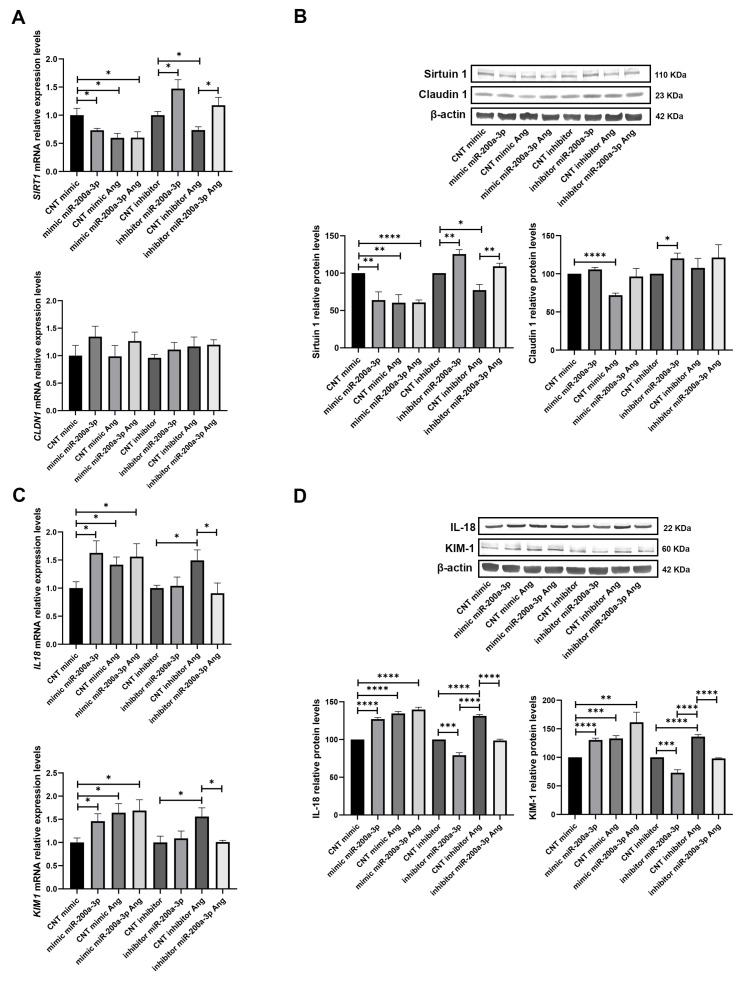
Effect of miR-200a-3p mimic and inhibitor on its miRNA target SIRT1 and in tubular injury under Ang II treatment: (**A**) SIRT1 and CLDN1 mRNA levels in RPTEC cultures subjected to miR-200a-3p mimic and inhibitor experiments under Ang II treatment. (**B**) Western blot membranes and graphs showing protein levels of Sirtuin 1 and Claudin 1 under miR-200a-3p mimic and inhibitor and Ang II conditions. (**C**) Tubular injury markers IL18 and KIM1 mRNA levels in RPTEC cultures subjected to miR-200a-3p mimic and inhibitor experiments under Ang II. (**D**) Western blot membranes and graphs showing protein levels of IL-18 and KIM-1 under miR-200a-3p mimic and inhibitor and Ang II conditions. (Original images can be found in Figure S6) N = 5 biological replicates for each group. Ang: Angiotensin II; CNT: control. For the CNT mimic and CNT inhibitor groups, miRNA levels are normalised to 1. * *p* < 0.05; ** *p* < 0.01, *** *p* < 0.001, and **** *p* < 0.0001 vs. the CNT mimic and CNT inhibitor group.

Under Ang II treatments, SIRT1 displayed similar results as obtained with glucose, with a decrease in mRNA levels in mimic condition (−1.37 FC, *p* < 0.05), when treated only with Ang II (−1.68 FC, *p* < 0.05), and with both the mimic and treatment (−1.66 FC, *p* < 0.05). The inhibitor of miR-200a-3p induced an increase in SIRT1 levels (1.47 FC, *p* < 0.05) (Figure 5A). Similarly, protein levels were decreased with the miR-200a-3p mimic, under treatment, and under both conditions (−1.56 FC, *p* < 0.01; −1.66 FC, *p* < 0.01; −1.64, *p* < 0.0001, respectively). With the inhibitor, we observed an increase (1.26 FC, *p* < 0.01) and also a compensation with the Ang II treatment (Figure 5B). On the other hand, CLDN1 mRNA levels under Ang II treatments did not show any changes, protein levels showed a decrease (−1.39 FC, *p* < 0.0001; Figure 5A), and only with the inhibitor condition were the levels increased (1.20 FC, *p* < 0.05; Figure 5B). Moreover, mRNA levels of IL18 were increased with the mimic, Ang II, and both conditions (1.63 FC, *p* < 0.05; 1.42 FC, *p* < 0.05; 1.56, *p* < 0.05, respectively). The inhibitor exerted a reduction in damage to CNT levels (Figure 5C). Similarly, KIM1 mRNA levels displayed an increase with the mimic, Ang II, and the combination of both (1.46 FC, *p* < 0.05; 1.64 FC, *p* < 0.05; 1.69, *p* < 0.05, respectively), and the inhibitor reduced the injury (Figure 5C). Protein levels of both injury markers displayed an increase under the mimic, Ang II treatment, and both conditions (1.27 FC, *p* < 0.0001; 1.35 FC, *p* < 0.0001; 1.40, *p* < 0.0001, respectively, for IL-18; and 1.31 FC, *p* < 0.0001; 1.33 FC, *p* < 0.001; 1.61, *p* < 0.01, respectively, for KIM-1). The inhibition of miR-200a-3p caused a reduction in both markers of damage (−1.26 FC and −1.21 FC, respectively; *p* < 0.001 for both) (Figure 5D).

## 4. Discussion

Renal damage is a critical complication of various pathologies, including DM and HTN. The renal tubules are particularly susceptible to injury, so understanding the molecular mechanisms underlying tubular damage and the search for novel and non-invasive biomarkers is crucial for developing effective therapeutic strategies to mitigate renal injury and preserve kidney function.

In this study, we show increased levels of miR-200a-3p in urinary EVs derived from patients with increased UAE and a direct correlation with this clinical parameter. Further analysis also evidences a good predictive power of miR-200a-3p to discriminate UAE presence. In a previous study we showed that this miRNA was increased in urinary pellets from patients with HTN and DM-associated renal damage [22]. Our current findings replicate these results in urinary EVs from a larger cohort of these patients. This evidences that urinary EVs are good transmitters of pathological states of renal cells, which point them as potential biomarkers of the cellular status. Importantly, the good predictive power of miR-200a-3p of UAE underscores its diagnostic potential for renal damage, and its non-invasive detection in urinary EVs further supports its value as a promising biomarker for clinical monitoring of renal injury. Current studies highlight the increasing value of EVs miRNAs as liquid biopsy biomarkers, and novel technologies are being developed for their measurement as an accurate and reliable method for clinical application [34]. For instance, Yang et al. developed a novel microfluidic cationic lipoplex nanoparticle (mCLN) assay designed for the rapid and sensitive detection of exosomal RNAs as liquid biopsy biomarkers for cancer. Using non-small cell lung cancer (NSCLC) as a model, the assay successfully detected exosomal miR-21 and TTF-1 mRNA with high sensitivity, requiring as little as 1 μL of serum. The mCLN assay outperformed conventional qRT-PCR by offering faster results (10 min vs. 4 h), higher diagnostic accuracy, and significantly reduced sample volume (30 μL vs. 100 μL) [35]. These findings highlight the assay’s strong potential as an efficient, minimally invasive diagnostic tool for disease detection and emphasise how microfluidics-based technologies have optimised the manner of EV miRNA detection and clinical applicability.

Moreover, we have deepened the study of miR-200a-3p and its target SIRT1 in an in vitro model of the disease in renal proximal tubular cells and their derived EVs, mimicking those conditions to which cells are subjected in HTN and DM-related renal injury. We observed an increase in miR-200a-3p in RPTECs and derived EVs and a decrease in SIRT1 under treatments with HG and Ang II. Previous researchers have reported that miR-200a-3p expression is linked to a reduction in SIRT1, identifying SIRT1 as its target [36,37]. The involvement of SIRT1 in kidney-related diseases is notable [38,39]. In renal disorders, SIRT1 enhances cell survival in damaged kidneys, helps regulate blood pressure, prevents cellular apoptosis in renal tubules, and triggers autophagy [40,41,42]. When SIRT1 expression decreases, diverse pathways such as the NF-κB signalling axis or TGF-β1 pathway are activated or inhibited, resulting in cell apoptosis, fibrosis, inflammation, and oxidative stress [43,44,45,46,47]. Interestingly, in our cellular model, although SIRT1 expression was reduced at early time points under HG conditions, a moderate increase was observed at 72 h. This apparent contradiction may reflect a biphasic or adaptive cellular response to sustained metabolic stress. It is plausible that, during prolonged exposure to HG, which comprises a stress situation, compensatory mechanisms such as the restoration of NAD^+^ levels or the activation of stress-responsive transcription factors, including PGC-1α and FOXO3a, promote a delayed upregulation of SIRT1 in an attempt to maintain cellular homeostasis [38,48,49]. In our previous study we observed an inverse relationship between SIRT1 and UAE levels in patients and a reduction in podocyte cultures when subjected to HG and Ang II treatments [22]. Our current findings in tubular cells reinforce the previous results and indicate opposite trends between miR-200a-3p and SIRT1 under damage presence, evidenced by a decrease in tubular markers E-cadherin and Aquaporin 1 and an increase in tubular injury markers IL18 and KIM1 under treatments. Previous studies by Hasegawa et al. observed the relationship between SIRT1 and CLDN1 through epigenetic mechanisms in podocytes [24]. Closer examination of the data presented in Figure 3 reveals that, while miR-200a-3p is upregulated and SIRT1 downregulated under both HG and Ang II conditions, *CLDN1* mRNA expression decreases only under HG, and CLDN1 protein levels even increase under Ang II. These apparent discrepancies suggest that the regulation of CLDN1 is not solely dependent on the miR-200a-3p/SIRT1 axis and is likely influenced by additional signalling pathways that act in a stimulus-specific manner. For instance, Ang II may activate renin–angiotensin system-related mechanisms that override or interact differently with the SIRT1–CLDN1 regulatory axis. Furthermore, the mismatch between mRNA and protein levels of CLDN1 under Ang II indicates potential post-transcriptional or post-translational regulation.

We subsequently developed functional studies on these cells to investigate the impact of miR-200a-3p overexpression and inhibition on its target SIRT1, CLDN1, and on tubular injury when combined with HG and Ang II treatments. The overexpression of miR-200a-3p led to decreased levels of its target SIRT1, a decrease in CLDN1 under HG but not under Ang II, and an increase in tubular injury measured by IL18 and KIM1. In contrast, inhibition of miR-200a-3p leads to an upregulation of SIRT1 under both treatment conditions. While CLDN1 expression remains unchanged at the mRNA level, it is notably increased at the protein level. Additionally, cellular damage is attenuated. These findings suggest that miR-200a-3p influences SIRT1 expression and that the inhibitor of this miRNA is able to increase the SIRT1 levels also when subjected to stress damage, underscoring the significant role of miR-200a-3p regulation over SIRT1 in tubular cells. Although our study primarily focused on SIRT1 due to our previous results [22] and its known relevance in renal protection, we recognise that miR-200a-3p likely regulates additional targets that may contribute to the observed phenotype. For example, KEAP1, a key inhibitor of NRF2-mediated antioxidant response, has been shown to be directly downregulated by miR-200a-3p, enhancing cellular resistance to oxidative stress [36,50]. Additionally, miR-200a-3p targets ZEB1 and ZEB2, transcription factors implicated in epithelial–mesenchymal transition (EMT) in renal tubular cells [51]. Increasing evidence from both in vitro and in vivo studies indicates that Sirt1 activity contributes to kidney protection [38,52,53,54], and its inhibition contributes to nephrotoxicity [55]. Consequently, other authors emphasise the potential therapeutic benefits of SIRT1 activation or overexpression in reducing kidney injury [56,57]. For instance, the use of morusin in STZ-induced diabetic mice alleviated tubulointerstitial damage and inhibited renal inflammation and fibrosis by upregulating SIRT1 expression [58]. Guo et al. investigate how Sirt1 activation can prevent the ACE2 downregulation by modulating the TIMP3/ADAM17 pathway in renal tubular cells under high-glucose conditions, which helps maintain ACE2 activity, crucial for renal protection in diabetic kidney disease [59]. Therefore, modifying the expression of SIRT1 through miR-200a-3p modulation could serve as a potential therapeutic target to promote renoprotection in HTN and DM-associated renal disease.

This study has limitations that warrant acknowledgement. It was carried out in a cell culture of RPTECs, which entails the simplicity of the environment compared to the behaviour in the whole organism. For this reason, further research could also be carried out using co-cultures and establish a stable cell line with miR-200a-3p inhibition to deepen the study of the interactions established between the renal cell lines and the role of miR-200a-3p long term. Moreover, miR-200a-3p is known to regulate additional targets implicated in injury-associated pathways, which may contribute to the effects observed in renal cells.

## 5. Conclusions

Collectively the findings of this study underpin miR-200a-3p role in renal injury in both EV urinary samples from patients and in an in vitro tubular cell disease model. miR-200a-3p inhibition enhances SIRT1 expression and reduces tubular injury, which evidences its promising potential as therapeutic target in renal damage associated with HTN and DM.

## Figures and Tables

**Table 1:** Clinical characteristics of study patients.

Variables	Non-Diabetic		Diabetic	
	Non-UAE (*n* = 19)	Increased UAE (*n* = 14)	Non-UAE (*n* = 9)	Increased UAE (*n* = 27)
Age (years)	54.45 ± 6.04	50.79 ± 9.26	54.78 ± 4.15	63.96 ± 10.99 †‡‡‡
Gender (male, %)	60	64.3	77.8	81.5
BMI (kg/m^2^)	30.6 ± 6.49	29.46 ± 5.01	29.51 ± 4.66	34.01 ± 7.05 ‡
Obesity (%)	40	42.9	44.4	63
SBP (mmHg)	134.05 ± 18.55	130.79 ± 10.59	140.78 ± 34.06	141.33 ± 21.56 ‡
DBP (mmHg)	88.00 ± 13.53	82.5 ± 8.86	89.00 ± 18.04	82.74 ± 11.70
Smoking (%)	40	57.1	55.6	22.2
Glucose (mg/dL)	103.65 ± 9.51	97.43 ± 17.46	149.11 ± 59.53 §§	149.33 ± 51.76 ‡‡‡
Glycated Hb (%)	3.37 ± 2.83	0.82 ± 2.09 *	6.49 ± 1.06 §§	7.09 ± 1.14 ‡‡‡
Plasma Cr (mg/dL)	0.85 ± 0.17	0.91 ± 0.34	0.99 ± 0.28	1.17 ± 0.59
GFR (mL/min/1.73 m^2^)	89.58 ± 16.97	92.04 ± 29.28	82.81 ± 23.51	78.95 ± 31.61
Ratio UAE/Cr (mg/g)	4.84 ± 6.45	126.67 ± 203.4	3.02 ± 1.48	361.97 ± 252.13 †††‡‡
Dyslipidaemia (%)	75	85.7	100	96.3
T Cholesterol (mg/dL)	184 ± 22.66	204.86 ± 35.98	150.67 ± 25.18	172.59 ± 35.32
LDL (mg/dL)	116.85 ± 19.74	133.43 ± 30.13	86.00 ± 20.58 §	104.22 ± 30.69 ‡‡
HDL (mg/dL)	51.30 ± 10.85	56.00 ± 13.80	43.67 ± 8.60	43.56 ± 11.18 ‡‡
TG (mg/dL)	121.40 ± 57.47	113.86 ± 44.12	159.00 ± 65.02	214.89 ± 177.86 ‡

Categorical variables expressed as percentages (%), quantitative variables as means ± SE. BMI, body mass index; Cr, creatinine; DBP, diastolic blood pressure; GFR, glomerular filtration rate; Hb, haemoglobin; HDL, high-density lipoprotein; LDL, low-density lipoprotein; SBP, systolic blood pressure; T Cholesterol, total cholesterol; TG, triglyceride; and UAE, urinary albumin excretion. Comparisons between non-diabetic groups: * *p* < 0.05. Comparisons between diabetic groups: † *p* < 0.05; †† *p *< 0.01; ††† *p* < 0.001. Comparisons between increased UAE groups: ‡ *p* < 0.05, ‡‡ *p* < 0.01, ‡‡‡ *p* < 0.001. Comparisons between No UAE groups: § *p* < 0.05, §§ *p* < 0.01. Comparisons between categorical variables performed using Fisher exact test. Comparisons between quantitative variables analysed for normality, Student *t*-test applied for normal variables, and Mann–Whitney test for non-normally distributed variables.

## Data Availability

All the data supporting the reported results are contained within this article and its Supplementary Material.

## Supplementary Materials

The following supporting information can be downloaded at https://www.mdpi.com/article/10.3390/biom15070995/s1 and at Figshare repository: https://figshare.com/s/deba8a13305c87a82501. Table S1: Primers used in the RT-qPCR for mRNA analysis. Figure S1: Characterisation of RPTEC lines and EVs derived from cell culture media; Figure S2: Whole membranes for Figure 2; Figure S3: Whole membranes for Figure 3; Figure S4: Verification of transfection efficiency and quantification of miR-200a-3p levels in RPTEC after mimic and inhibitor transfections and treatments with glucose and Ang II; Figure S5: Whole membranes for Figure 4; Figure S6: Whole membranes for Figure 5; Figure S7: Whole membranes for Figure S1.

## Abbreviations

The following abbreviations are used in this manuscript:

ACR:	Albumin/creatinine ratio
ACTB	β-Actin
AQP1	Aquaporin 1
AUC	Area under the curve
Ang II	Angiotensin II
B2MG	β2-microglobulin
BSA	Bovine serum albumin
CDH1	E-cadherin
CI	Confidence interval
CLDN1	Claudin 1
CNT	Control
DBP	Diastolic blood pressure
DM	Diabetes mellitus
DMEM	Dulbecco’s Modified Eagle Medium
DTT	Dithiothreitol
EV	Extracellular vesicle
EXO-U	Exosome derived from patient urine
FC	Fold change
FBS	Foetal bovine serum
GFR	Glomerular filtration rate
hEGF	Human epidermal growth factor
HG	High glucose
HTN	Hypertension
ITS	Insulin transferrin selenium
miRNAs	MicroRNAs
NAD	Nicotinamide adenine dinucleotide
NG	Normal glucose
PBS	Phosphate-buffered saline
PVDF	Polyvinylidene difluoride
P/S	Penicillin/streptomycin
UAE	Urinary albumin excretion
ROC	Receiver Operating Characteristic
RPTEC	Renal proximal tubular epithelial cell
RT	Room temperature
RT-qPCR	Quantitative Real-Time Polymerase Chain Reaction
SEM	Standard error of the mean
SIRT 1	Sirtuin 1
SBP	Systolic blood pressure
TBS-T	Tris-buffered saline with Tween
7-AAD	7-amino-actinomycin D
