# Supplementary material for: Inhibiting miR-200a-3p Increases Sirtuin 1 and Mitigates Kidney Injury in a Tubular Cell Model of Diabetes and Hypertension-Related Renal Damage
Source: Biomolecules. 2025 Jul 11;15(7):995. doi: 10.3390/biom15070995 (PMC12292945; doi:10.3390/biom15070995)
Supplement: Supplementary file 1 [file biomolecules-15-00995-s001.zip › biomolecules-3700555-supplementary.pdf]

# Inhibiting miR-200a-3p increases Sirtuin 1 and mitigates kidney injury in a tubular cell model of diabetes and hypertension-related renal damage

**Olga Martinez-Arroyo [1,+], Ana Flores-Chova [1,+], Marta Mendez-Debaets [1], Laia Garcia-Ferran [1], Lesley Escriva [1], Maria J Forner [1,2,3], Josep Redon [1,4], Raquel Cortes [1,*] and Ana Ortega [1,5,*]**

**SUPPLEMENTARY MATERIAL**

**Table S1. Primers used in the RT-qPCR for mRNA analysis**

| GENE | FORWARD SEQUENCE 5′→3′ | REVERSE SEQUENCE 3′→5′ | AMPLICON SIZE | Tm (°C) |
|---|---|---|---|---|
| *CDH1* | GTGAACAC-CTACAATGCCGC | GAAACTCTCTCGGTCCAGCC | 138 bp | 62 °C |
| *AQP1* | TATGCGTGCTGGC-TACTACCG | CACCCAGAAAATCCAG-TGGTTG | 191 bp | 60 °C |
| *SIRT1* | TTGTTATTGGGTCTTCCCTCAAA | AAATGCAGATGAGGCAAAGGTT | 112 bp | 62 °C |
| *CLDN1* | CCGTTGGCATGAAGTG-TATG | AGCCAGACCTGCAAGAA-GAA | 101 bp | 60 °C |
| *IL18* | GCTGAA-GATGATGAAAACCTGGA | AATAGAGGCCGAT-TTCCTTGGT | 119 bp | 64 °C |
| *KIM1* | CAGAAACCCACCCTAC-GACA | GGTGTCATTCCCATCTGTTGTG | 92 bp | 64 °C |
| *ACTB* | TGGAGAAAATCTGGCAC-CAC | CATGATCTGGGTCATCTTCTCG | 125 bp | 62 °C |
| *B2MG* | TCCAGCGTACTCCAAA-GATTC | GTCAACTTCAATGTCG-GATGG | 113 bp | 62 °C |

CDH1: E-cadherin AQP1: Aquaporin 1; SIRT1: Sirtuin-1; CLDN1: Claudin-1; KIM-1: Kidney injury molecule-1; IL18: Interleukin-18; ACTB: actin beta gene; B2MG: Beta-2-Microglobulin; bp: base pairs; Tm: Melting Temperature

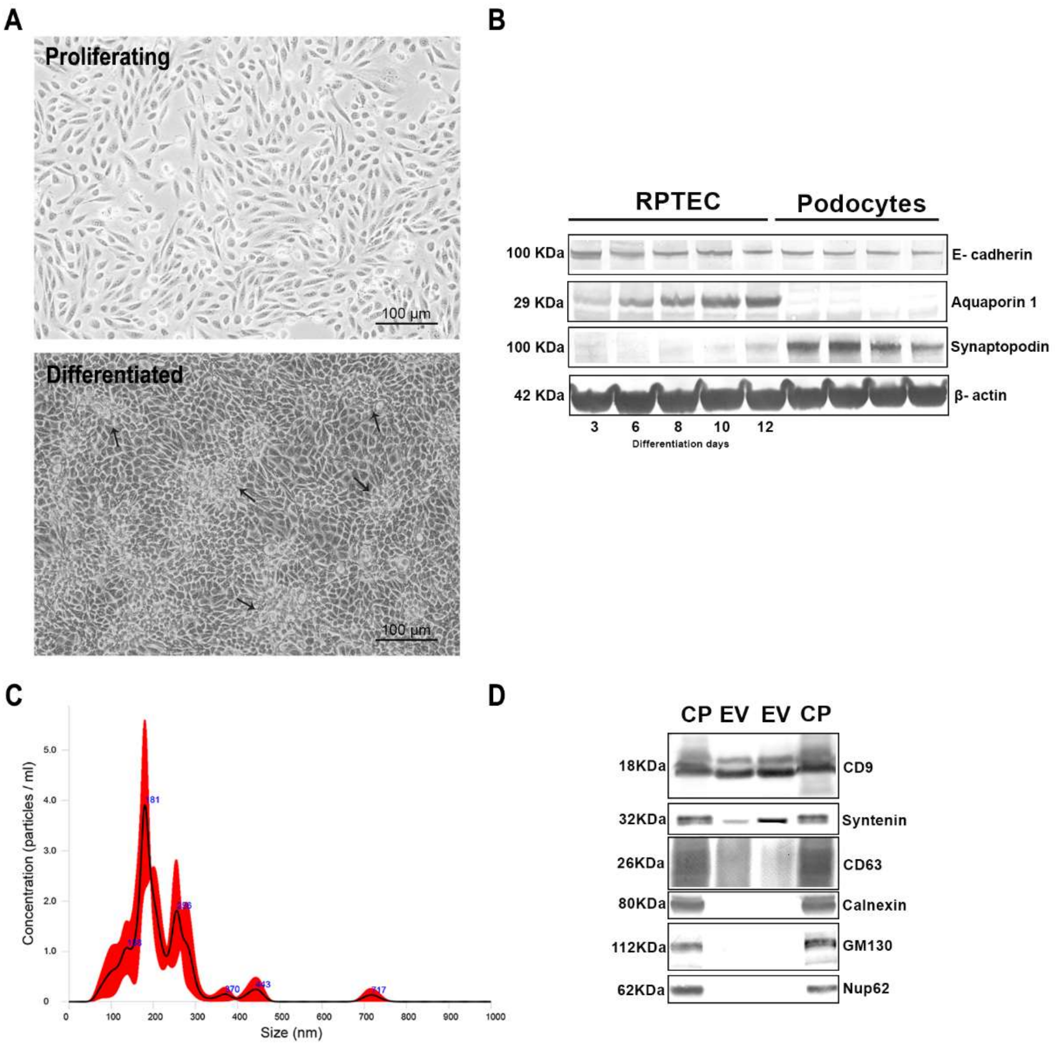


**Figure S1**. Characterisation of RPTEC lines and EVs derived from cell culture media. **A.** Optical microscopy images of proliferating and differentiated RPTECs showing RPTEC cobblestone morphology during proliferation phase and the characteristic dome formation in differentiated state (black arrows). **B**. Western blot membranes of RPTEC at different stages of differentiation and podoytes displaying the protein levels of RPTEC (E-cadherin and Aquaporin 1) and podocyte (Synaptopodin) markers, showing the specificity of the expression in RPTEC cell line. **C.** Nanoparticle Tracking Analysis (NTA) graph of EVs derived from RPTEC cell culture media showing the EV concentration and size with a peak between 140-200 nm. **D.** Western blot membranes of cell pellet (CP) and EVs showing the presence of EV characteristic markers CD9, Syntenin and CD63 and the absence of signal of the organelle markers calnexin (endoplasmic reticulum), GM130 (Golgi apparatus) and Nup62 (nucleus).

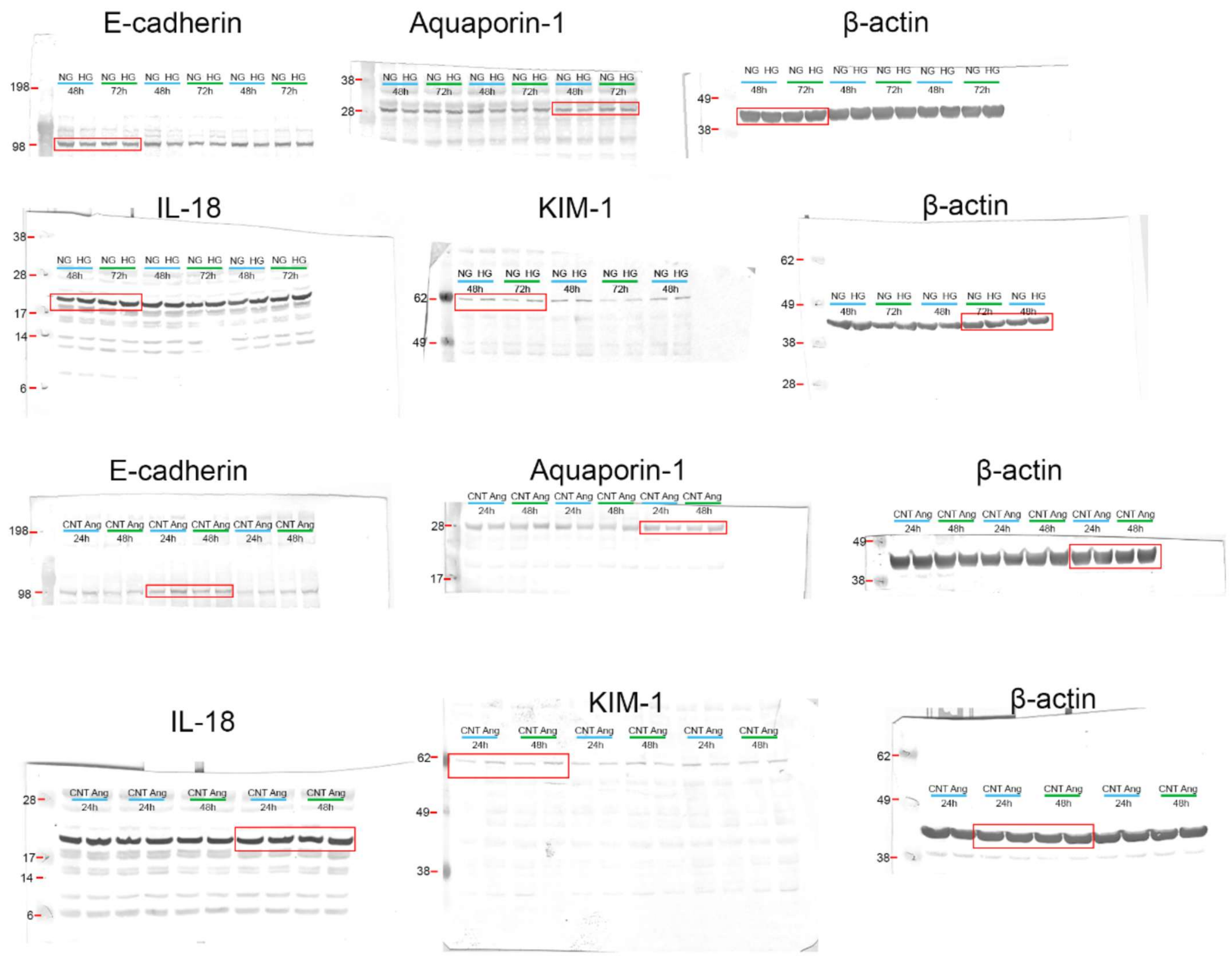


**Figure S2.** Whole membranes for Figure 2. **Ang:** Angiotensin II**;** **CNT:** control**;** **NG:** normal glucose**;** **HG:** high glucose

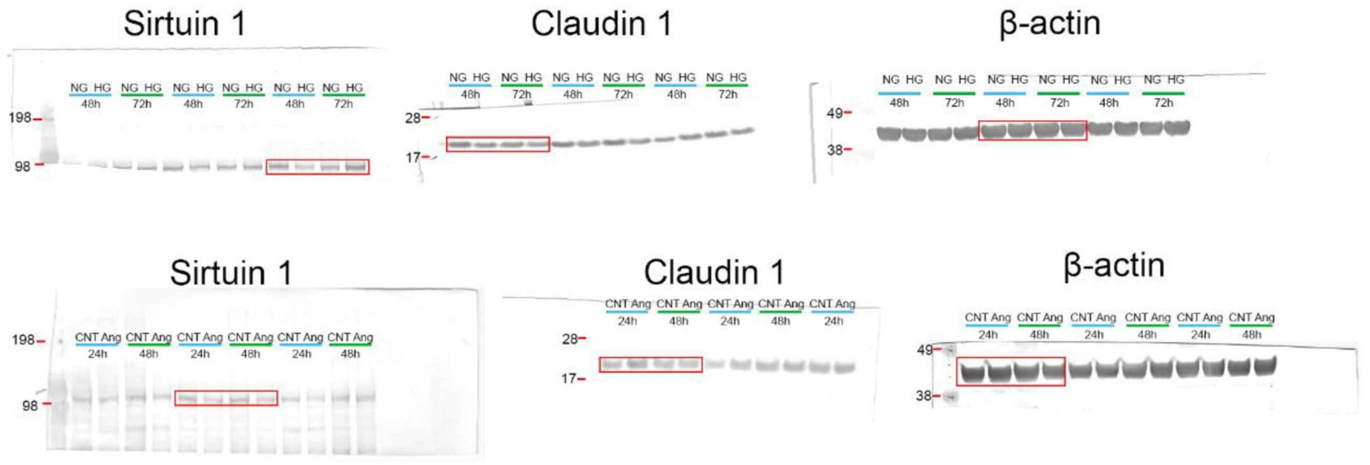


**Figure S3.** Whole membranes for Figure 3 **Ang:** Angiotensin II**;** **CNT:** control**;** **NG:** normal glucose**;** **HG:** high glucose

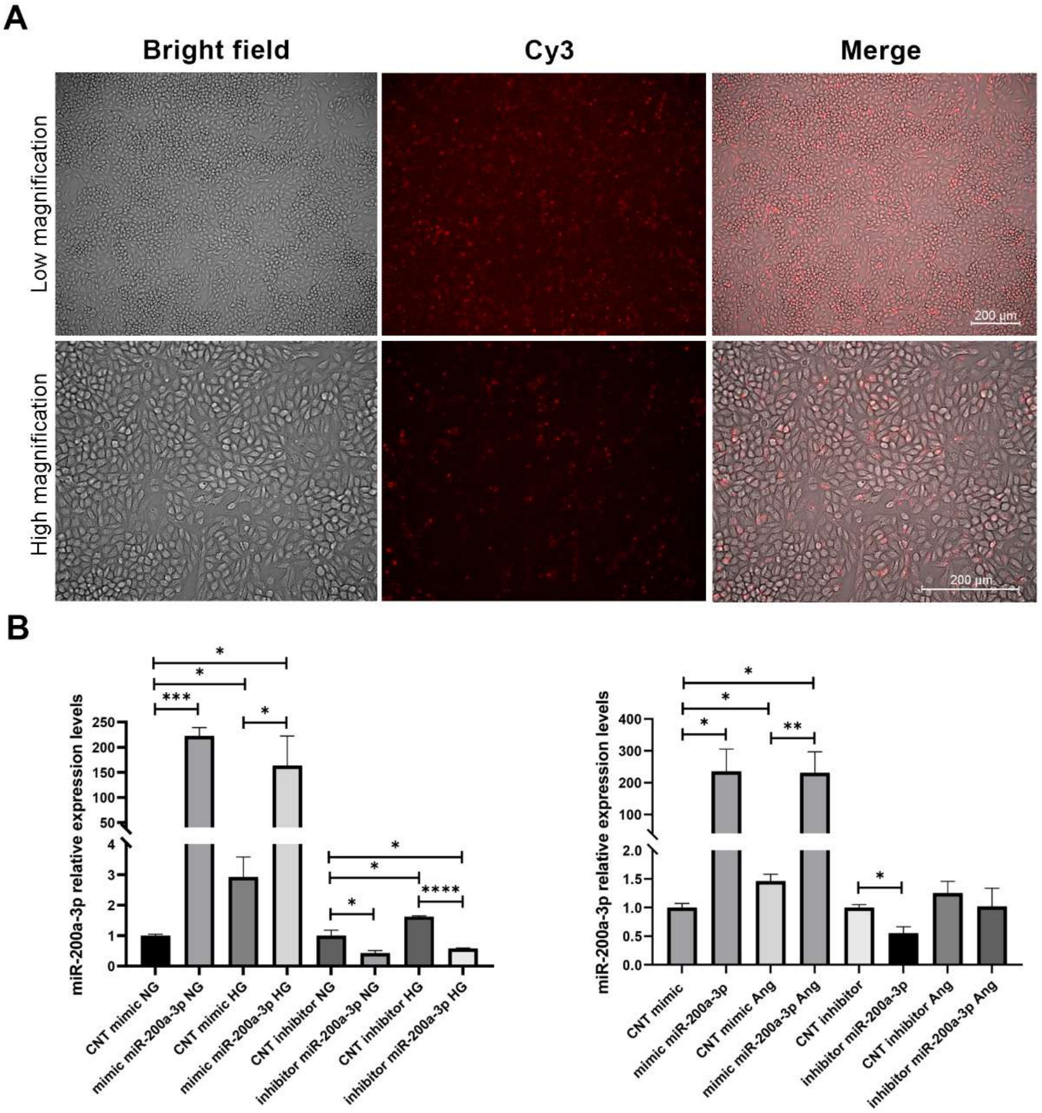


**Figure S4.** Verification of transfection efficiency and quantification of miR-200a-3p levels in RPTEC after mimic and inhibitor transfections and treatments with glucose and Ang II. **A**. Microscopy images at low and high magnifications of bright field and fluorescence of Cy3 labeling in RPTECs showing the internalization of the dye inside cells. **B.** miR-200a-3p levels after transfection and under glucose treatments (left) and miR-200a-3p levels after transfection and under Ang II treatments (right). N = 5 for each group. Ang II: Angiotensin II; CNT: control; NG: normal glucose; HG: high glucose. For the CNT mimic and CNT inhibitor groups, miRNA levels are normalised to 1. *p<0.05; ***p<0.001.

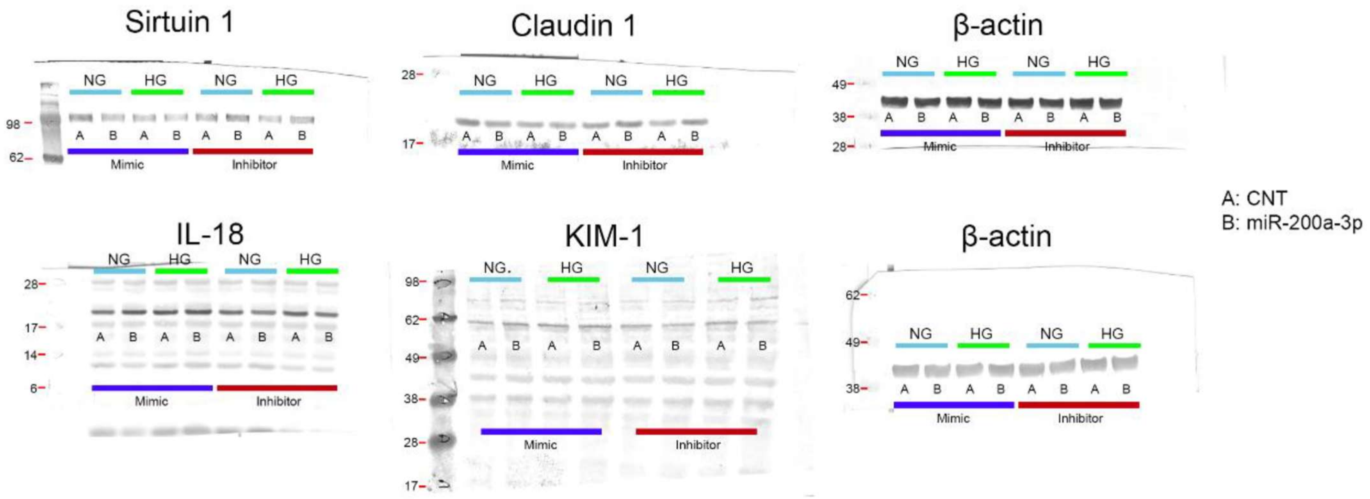


**Figure S5.** Whole membranes for Figure 4 **NG:** normal glucos**e; HG:** high glucose

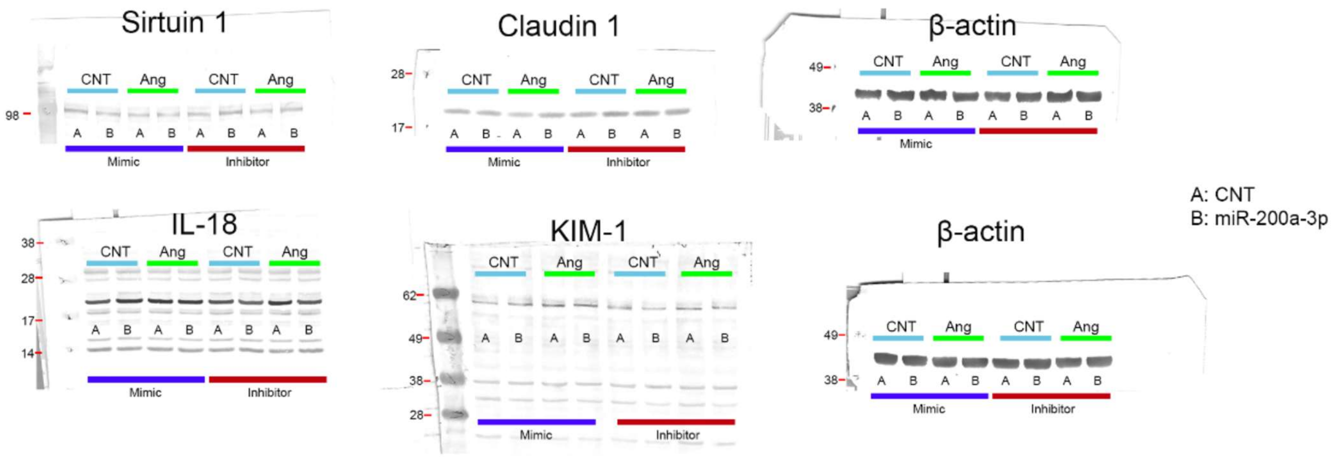


**Figure S6.** Whole membranes for Figure 5 **Ang:** Angiotensin II**; CNT:** control**; NG:** normal glucos**e; HG:** high glucose

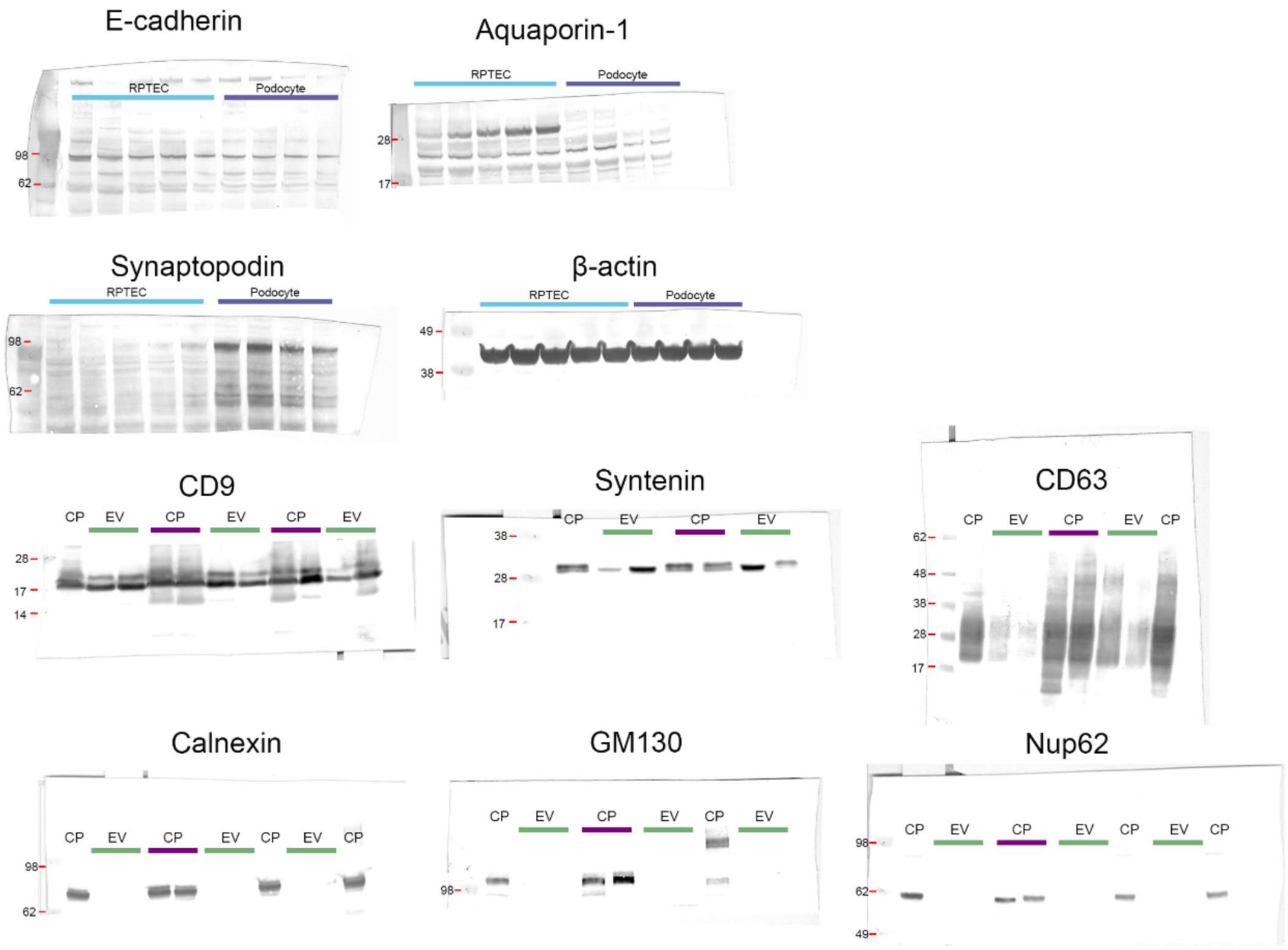


**Figure S7.** Whole membranes for Figure S1
